# Treatment of hepatocellular carcinoma with major portal vein thrombosis by combined therapy with subcutaneous interferon-*α* and intra-arterial 5-fluorouracil; role of type 1 interferon receptor expression

**DOI:** 10.1038/sj.bjc.6602742

**Published:** 2005-08-16

**Authors:** H Ota, H Nagano, M Sakon, H Eguchi, M Kondo, T Yamamoto, M Nakamura, B Damdinsuren, H Wada, S Marubashi, A Miyamoto, K Dono, K Umeshita, S Nakamori, K Wakasa, M Monden

**Affiliations:** 1 Department of Surgery and Clinical Oncology, Graduate School of Medicine, Osaka University, 2-2, Yamadaoka E-2, Suita, Osaka 565-0871, Japan; 2 Department of Pathology, Osaka City University Hospital, 1-5-7, Asahi-cho Abeno-ku, Osaka 545-0051, Osaka, Japan

**Keywords:** hepatocellular carcinoma, IFNAR2, portal vein thrombosis, arterial infusion chemotherapy

## Abstract

We previously reported the beneficial effects of combination therapy of interferon (IFN)-*α*/5-fluorouracil (FU) for advanced hepatocellular carcinoma (HCC) with tumour thrombi in the major portal branches. This report describes the results of longer follow-up and includes more than double the number of patients relative to the original report, and evaluates the role of IFN-*α*/type 2 interferon receptor (IFNAR2) expression on the response to the combination therapy. The study subjects were 55 patients with advanced HCC and tumour thrombi in the major branches of the portal vein (Vp3 or 4). They were treated with at least two courses of IFN-*α*/5-FU without major complication. In the 55 patients, 24 (43.6%) showed objective response (eight (14.5%) showed complete response, 16 (29.1%) partial response), four (7.3%) showed no response, and 27 (49.1%) showed progressive disease. Immunohistochemically, IFNAR2 expression was detected in nine out of 13 (69.2%) patients. There was significant difference in the time-to-progression survival (*P*=0.0002) and the overall survival (*P*<0.0001) between IFNAR2-positive and -negative cases. There was a significant correlation between IFNAR2 expression and response to IFN-*α*/5-FU combination therapy in univariate analysis (*P*=0.0070). IFN-*α*/5-FU combination therapy is a promising modality for advanced HCC with tumour thrombi in the major portal branches and could significantly depend on IFNAR2 expression.

The prognosis of patients with advanced hepatocellular carcinoma (HCC) remains poor, particularly in patients with tumour thrombi in the major branches of the portal vein (Vp3 or Vp4) (Chen *et al*, 1994; Yen *et al*, 1995; Tanaka *et al*, 1996; Ando *et al*, 1997; Furuse *et al*, 1997; Lee *et al*, 1997; CLIP investigators, 1998; Asahara *et al*, 1999; Yamakado *et al*, 1999). Almost all patients with unresectable tumours die within several months and have poor quality of life (QOL) due to intractable ascites or oesophageal bleeding. Even in patients with resectable HCC, the prognosis is extremely poor despite aggressive surgery (Tanaka *et al*, 1996; Asahara *et al*, 1999). In such a situation, conventional therapies like percutaneous ethanol injection, microwave coagulation therapy, and transcatheter arterial embolisation generally have no clinical effect on HCC with portal tumour thrombi due to poor efficacy and possible complications (Chen *et al*, 1994; Yen *et al*, 1995; Furuse *et al*, 1997). Furthermore, arterial infusion chemotherapy has also been attempted, but its effectiveness is still unsatisfactory (Doci *et al*, 1988; Ando *et al*, 1997). Therefore, a new strategy is required for these patients with intractable HCC and tumour thrombi in the major branch of the portal vein.

Several recent studies have indicated the beneficial effects of interferon (IFN)-*α*-based combination therapies for hepatomas. Patt *et al* (1993) reported 31% response rate in patients with unresectable advanced hepatoma and low alpha-fetoprotein (AFP) levels. Using intra-arterial infusion chemotherapy and systemic IFN-*α*, Urabe *et al* (1998) reported a response rate of 47% in patients with Vp3. In another study, Leung *et al* (1999) used cisplatin, doxorubicin, and IFN-*α* and reported that this treatment converted nine patients of 36 patients with inoperable HCC to become suitable for tumour resection, and none developed postoperative recurrence. More recently, Chung *et al* (2000) reported partial response in 33% (six out of 18) of their patients with major portal vein thrombosis or distant metastases, who received systemic combination therapy with IFN-*α* and cisplatin (CDDP). Based on this background, we experienced a patient with advanced HCC and lung metastasis, who was successfully treated with a combination therapy of IFN-*α* and tegafur/uracil (UFT) (Miyamoto *et al*, 2000). In addition, we recently reported the outstanding effects of intra-arterial 5-fluorouracil (5-FU) combined with subcutaneous IFN-*α* for advanced HCC with tumour thrombi in the major portal branches, since 1997 (Sakon *et al*, 2002). Based on our results, the marked effects and acceptable toxicity of our therapy in HCC patients with extremely poor prognosis suggest that IFN-*α*/5-FU combination therapy is a promising treatment regimen.

However, the response rate in the above studies was not absolute; to advance the effect of IFN-*α*/5-FU combination therapy and to increase the response rate, it is necessary to investigate the mechanism and to predict the response to IFN-*α*/5-FU combination therapy, for patients with advanced HCC. Interferon-*α* suppresses the proliferation of all type I interferon receptor 2 (IFNAR2)-positive cancer cell lines *in vitro* through mechanisms related to apoptosis or inhibition of cell cycle. Furthermore, the antineoplastic effects of IFN-*α* may be mediated through its high-affinity membrane type I receptor, IFNAR2. Thus, IFNAR2 expression in HCC tissues may be a useful predictor of the response to IFN-*α*/5-FU combination therapy.

The present study is an extension to our previous work (Sakon *et al*, 2002), in which we examined the clinical effects of the combination therapy of subcutaneous IFN-*α* and arterial infusion of 5-FU in 55 patients with HCC associated with Vp3 and investigated whether the response to such therapy is influenced by the expression level of IFNAR2.

## MATERIALS AND METHODS

### Patients and selection criteria

This was a single arm open label study, based on our pervious report (Sakon *et al*, 2002). Between December 1997 and December 2003, 124 patients with advanced HCC and tumour thrombi in the major branches of the portal vein and multiple liver metastasis (Vp3 or 4, IM3) were diagnosed in our department. Among these patients, 10 were excluded because of liver failure, 44 underwent hepatectomy before IFN-*α*/5-FU combination therapy because they were within the indications for operation, 15 received other treatments, seven had distant metastases, four were over 75 years of age, and four patients refused to sign the informed consent form because of fear of adverse effects. The remaining 55 patients were enrolled in the study. All patients were radiologically confirmed to have tumour thrombi in the major branches of the portal vein (Vp3 or 4), and multiple intrahepatic metastases (IM3). The diagnosis was based on liver function tests, serum alpha-fetoprotein (AFP), serum protein induced by vitamin K absence or antagonist-II (PIVKA-II) and imaging techniques including computed tomography (CT) scan, magnetic resonance imaging (MRI), hepatic angiography and arterial portography. The final diagnosis was unresectable HCC in 55 patients mainly because of marked portal tumour thrombi extending into all the three major branches and/or poor liver function. Among the 55 patients, 13 signed informed consent documents approved by the institutional review board attesting to the fact that they were aware of the investigative nature of the study. Therefore, liver biopsy could be performed in these 13 patients. On the other hand, the remaining 42 patients did not sign informed consent documents because of fear of tumour implantation and/or bleeding accompanying liver biopsy.

The following were the eligibility criteria for selection for intra-arterial combination therapy; (1) age of more than 20 years and less than 75 years, (2) tumour thrombi invading at least one of the main branches of the portal vein, (3) presence of multiple intrahepatic metastases in more than three segments (IM3), (4) absence of extrahepatic metastases, (5) a granulocyte count of more than 2500 *μ*l^−1^ and less than 12 000 *μ*l^−1^, (6) a red blood cell count of more than 8.0 g dl^−1^, (7) a platelet count exceeding 8 × 10^4^ *μ*l^−1^, (8) GOT and GPT of less than 100 IU l^−1^. (9) total bilirubin less than 1.4 g dl^−1^, (10) serum BUN less than 30 mg dl^−1^, (11) serum creatinine less than 1.5 mg dl^−1^, (12) successful implantation of intra-arterial catheter and drug delivery system, and (13) a performance status of level 0–2 (Eastern Cooperative Oncology Group, ECOG). These eligibility criteria were based on our previous report (Sakon *et al*, 2002). All patients signed informed consent documents approved by the institutional review board attesting to the fact that they were aware of the investigational nature of the study and were willing to try the combination therapy.

### Treatment protocol

In each of our 55 patients, an intra-arterial catheter was inserted through the subclavian or femoral artery with a subcutaneously implanted drug delivery system (Toyoda *et al*, 1995). Each patient was treated with subcutaneous IFN-*α* (OIF, Otsuka Pharmaceutical Co., Tokushima, Japan) and intra-arterial infusion of 5-FU (Kyowa Hakko Co., Tokyo). Interferon-*α* (5 × 10^6^ U (5 MU)) was administered on days 1, 3, and 5 of each week. Continuous infusion chemotherapy (5-FU, 300 mg m^−2^ day^−1^) through the proper hepatic artery was performed every 2 weeks for two sessions via a catheter connected to a subcutaneously implanted drug delivery system. In summary, 55 patients received this therapy for multiple HCCs with tumour thrombi in the main branch of the portal vein. There was no dose escalation, because none of the six patients, in whom the adverse effects reached level 2 of the ECOG classification, were there (with the exception of platelet and leukocyte counts of <0.4 × 10^5^ *μ*l^−1^ and 2000 *μ*l^−1^, respectively, before treatment because of cirrhosis).

### Evaluation of response to anticancer therapy

In addition to serum chemistry, tumour markers such as AFP and PIVKA-II were measured at least once every 4 weeks. Abdominal CT scan or dynamic MRI was also performed before and after treatment, and once every 3 months thereafter. We evaluated the effects of therapy at 3 months after the commencement of this therapy. Simon's two-stage design (WHO handbook, 1979) was used to calculate the sample size for clinical response evaluation. The objective response was classified according to the ECOG criteria (Oken *et al*, 1982). Complete response (CR) was defined as the normalisation of tumour markers and the disappearance of all tumours and portal vein thrombosis on CT and/or MRI. Partial response (PR) represented a decrease of tumour markers and 50–99% regression on the two-dimensional measurement. No change (NC) represented less than 50% regression or less than 25% progression. Progressive disease (PD) represented more than 25% progression. This classification was based on the World Health Organization (WHO) handbook (WHO handbook, 1979).

### Reagents

Rabbit polyclonal anti-human IFNAR2 antibody (OCT4813, Otsuka Pharmaceutical Co., Tokushima) and its blocking peptide were prepared according to the report by Novick *et al* (1994).

### Immunohistochemistry

The expression of IFNAR2 was examined in 13 tumour samples of 55 cases by immunohistochemistry (Figures 3, 4 and 5, Table 3). Biopsy samples were obtained with a needle guide/cover kit and a 16-gauge core tissue biopsy needle (Bard MAGNUM: C.R. Bard Inc., Covington, USA) under colour Doppler ultrasonography. Immunohistochemistry was carried out according to the method described previously by our laboratories (Kondo *et al*, 2000). Tissue sections (4-*μ*m thick) were deparaffinised in xylene and heat antigen retrieval was performed as described previously (Ciaparrone *et al*, 1998). The slides were then processed for immunohistochemistry on the TeckMate Horizon automated staining system (Dako Corporation, Carpinteria, CA), using the EnVision+ peroxidase kit (DAKO, Glostrup, Denmark). In the step of primary antibody reaction, the slides were incubated with the IFNAR2 antibody (final concentration: 2.5 mg ml^−1^) for 1 h at room temperature. For negative controls, nonimmunised rabbit IgG (Vector Laboratories, Burlingame, CA, USA) or TBS (Tris-buffered saline) was used as a substitute for the primary antibody to verify the possibility of false positive responses from nonspecific binding of IgG or from the secondary antibody. In addition, absorption tests were performed on tissue sections. The intensity of IFNAR2 was scored in a scale from 0 to 2. IFN-*α*/type 2 interferon receptor expression was often heterogeneous. The histological or immunohistological type that constituted the major volume of the tumour was selected as the representative type. Staining was repeated at least twice to avoid possible technical errors but basically identical results were obtained. All slides were interpreted by one of two investigators (HO or MK) in a blinded manner without knowledge of the clinical and pathological parameters. When the initial diagnosis was different, the final diagnosis was mutually determined using a multihead microscope by two investigators (HO or MK and KW).

### Statistical analysis

The Breslow–Gehan–Wilcoxon univariate test was used to examine the possible relationship between the effect of therapy (CR, PR *vs* NC, PD), Child–Pugh score, serum AFP, serum PIVKA-II, Okuda score, CLIP score (CLIP investigators, 1998) and the expression of IFNAR2. Survival curves were constructed using the Kaplan–Meier method. Differences in distribution between groups were compared by the *χ*^2^ test and differences in mean values by the Student's *t*-test. All data were expressed as mean±s.e.m. A *P*-value less than 0.05 denoted the presence of a statistically significant difference.

## RESULTS

### Patient characteristics

Between 1997 and 2003, 55 patients who had HCC with tumour thrombi in the major branches of portal vein were enrolled in this study. The clinical characteristics of the study participants are summarized in Table 1. The median age of patients was 58.2 years. In total, 24 patients were positive for HBV (anti-HBs antigen), while 29 were positive for HCV (anti-HCV antibody) and five patients were negative for both HBV and HCV. The mean granulocyte count was 5310 *μ*l^−1^, the mean platelet count was 15.3 × 10^4^ *μ*l^−1^. Almost all cases were Child A or B. Alpha-fetoprotein and/or PIVKA-II were abnormal in 52 of 55 cases. The median tumour size was 6.4 cm.

### Clinical response to combination therapy

**Figure 1:**
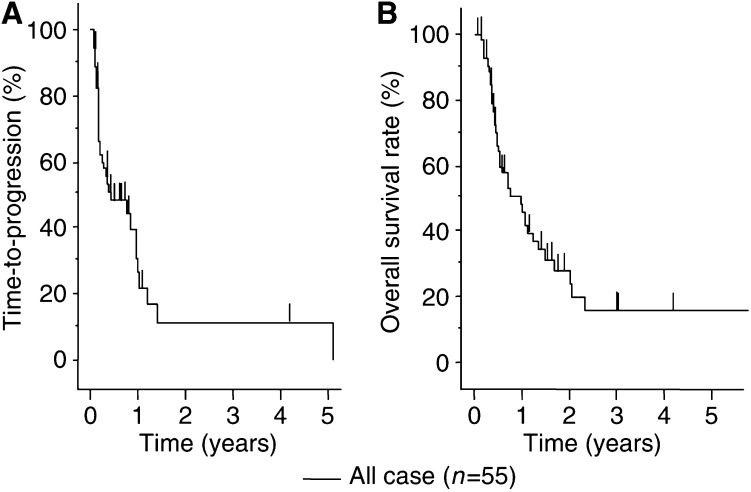
Kaplan–Meyer analysis for efficiency of IFN-*α*/5-FU combination therapy. All 55 patients completed at least two courses of IFN-*α*/5-FU combination therapy. (**A**) With respect to time-to-progression, the median progression-free survival period was 5.2 months and the 1-, 2-, 3-, and 5-year progression-free survival rates were 11.3, 3.8, 3.8, and 1.9%, respectively. (**B**) With respect to overall survival, the median overall survival period was 11.8 months and the 1-, 2-, 3-, and 5-year survival rates were 48.9, 28.8, 16.4 and 16.4%, respectively.

**Figure 2:**
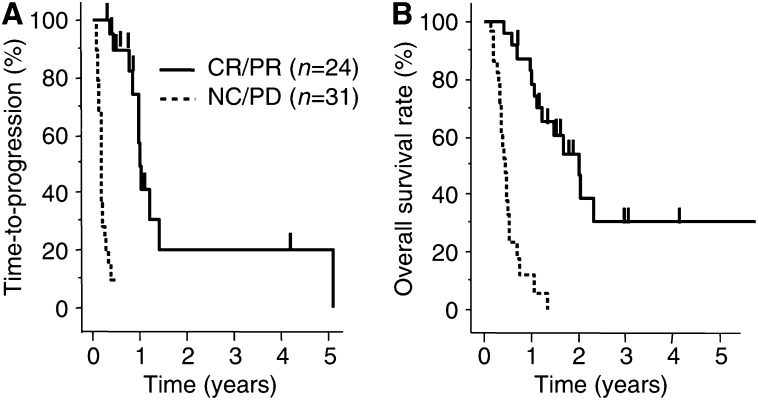
Kaplan–Meyer analysis for efficiency of IFN-*α*/5-FU combination therapy. All 55 patients completed at least two courses of IFN-*α*/5-FU combination therapy. A total of 24 (43.6%) patients were assessed as objective responders; eight (14.5%) complete responders (CR), 16 (29.1%) partial responders (PR), four (7.3%) nonresponder (NC), and 27 (49.1%) showed progressive disease (PD). With respect to time-to-progression and overall survival, there was a significant difference between objective responders (CR/PR) and nonresponders (NC/PD) (*P*<0.0001). (**A**) The median progression-free survival periods of CR/PR cases (*n*=24) was 12.0 months and that of NC/PD cases (*n*=31) was 2.2 months. The 1-, 2-, and 3-year progression-free survival rates of CR/PR cases were 49.3, 20.6, and 20.6%, respectively, and those of NC/PD cases were 0, 0, and 0%, respectively. (**B**) The median survival period of CR/PR cases (*n*=24) was 24.4 months and that of NC/PD cases (*n*=31) was 5.4 months. The 1-, 2-, and 3-year survival rates of CR/PR cases were 82.9, 54.2, and 30.9%, respectively, and those of NC/PD cases were 13.1, 0, and 0%, respectively.

In this study, 55 patients with unresectable HCCs completed at least two cycles of IFN-*α*/5-FU combination therapy, with a mean number of treatment cycles of 3.6 cycles (range, 2–12 cycles). With respect to time-to-progression, the median progression-free survival period was 5.2 months and the 1-, 2-, 3- and 5-year progression-free survival rates were 11.3, 3.8, 3.8, and 1.9%, respectively. Furthermore, the median overall survival period was 11.8 months and the 1-, 2-, 3- and 5-year survival rates were 48.9, 28.8, 16.4, and 16.4%, respectively. Of the 55 patients, 24 (43.6%) showed objective response, eight (14.5%) showed CR, 16 (29.1%) showed PR, four (7.3%) showed NC, 27 (49.1%) showed PD. The median progression-free survival periods of CR/PR cases (*n*=24) was 12.0 months and that of NC/PD cases (*n*=31) was 2.2 months. The 1-, 2-, and 3-year progression-free survival rates of CR/PR cases were 49.3, 20.6, and 20.6%, respectively, and those of NC/PD cases were 0, 0, and 0%, respectively. The median survival periods of CR/PR cases (*n*=24) was 24.4 months and that of NC/PD cases (*n*=31) was 5.4 months. The 1-, 2-, and 3-year survival rates of CR/PR cases were 82.9, 54.2, and 30.9%, respectively, and those of NC/PD cases were 13.1, 0, and 0%, respectively. The time-to-progression survival and overall survival curves are shown in Figures 1 and 2. There were significant difference in the time-to-progression survival and the overall survival between responders (CR/PR) and nonresponders (NC/PD) (*P*<0.0001).

### Adverse effects

None of the patients developed side effects related to catheter insertion or subcutaneous implantation of the drug delivery system. However, 14.6% of patients developed grade 3 leukopenia, thrombocytopenia, or anaemia, but drip transfusion of granulocyte colony-stimulating factors was not used during this study.

Nonhaematological toxicities included grade 1 or 2 fever (100% of patients), chilling sense (100%), flu-like syndrome (100%), generalised fatigue (25.4%), nausea (5.5%), diarrhoea (3.6%), gastric ulceration (1.8%), skin reaction (5.5%), and depression (3.6%). The side effects are summarised in Table 2.

### Correlation between IFNAR2 immunostaining pattern and prognosis

**Figure 3:**
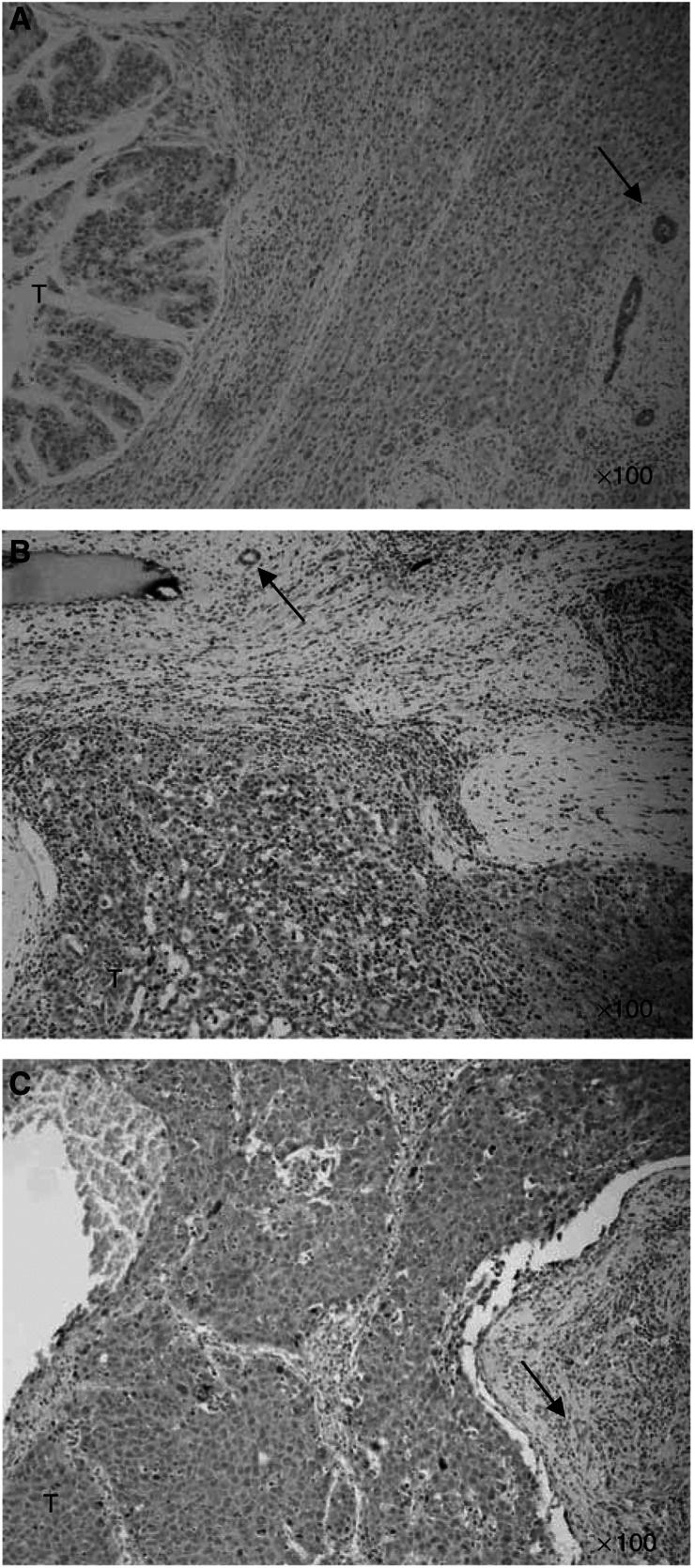
Immunohistochemical analysis of IFNAR2 expression in HCC tissues. The intensity of IFNAR2 was scored in a scale from 0 to 2; 0 representing no or faint staining (**A**); 1=moderate staining (**B**); and 2=strong staining (**C**). The latter level of staining was used as an inner control (arrow) within the sample, which was designated arbitrarily as intensity 1, because the epithelial cells of the bile ducts generally expressed moderate levels of IFNAR2.

**Figure 4:**
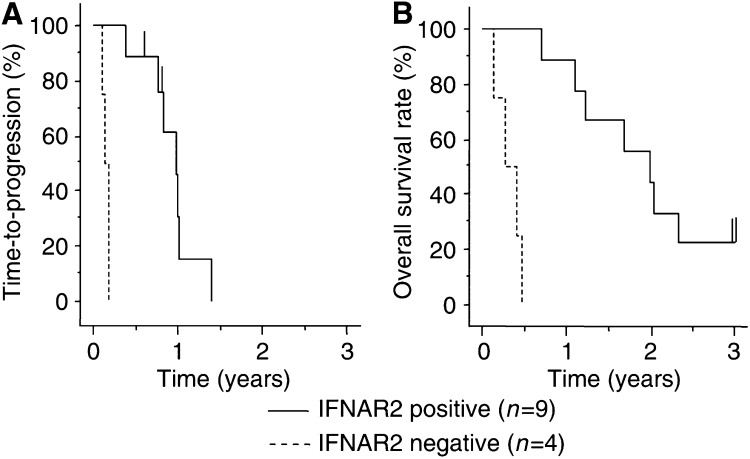
Kaplan–Meyer analysis for expression of IFNAR2. Immunohistochemical analysis was performed in 13 biopsy samples. The expression rate of IFNAR2 was 69.2% (nine out of 13). (**A**) The median progression-free survival rate was 12.6 months for IFNAR2-positive cases and 1.7 months for IFNAR2-negative cases. The 1-, 2-, and 3-year progression-free survival rates were 30.5, 0, and 0%, respectively, for IFNAR2-positive cases, 0, 0, and 0%, respectively, for IFNAR2-negative cases. There was a significant correlation between positive and negative cases (*P*=0.0002). (**B**) The median overall survival rate was 24.4 months for IFNAR2-positive cases and 3.4 months for IFNAR2-negative cases. The overall survival rates at 1, 2, and 3 years of IFNAR2-positive patients (*n*=9) (88.9, 55.6 and 22.2%, respectively) were significantly higher than the rates (0, 0, and 0%, respectively) of IFNAR2-negative patients (*n*=4). There was a significant difference between positive and negative cases (*P*<0.0001).

For each section, the intensity of IFNAR2 immunostaining was scored on a scale from 0 to 2 where 0 represented no or faint immunostaining (Figure 3A), 1: moderate (Figure 3B), and 2: strong staining (Figure 3C). IFN-*α*/type 2 interferon receptor expression was faint or undetectable in the vascular epithelium whereas epithelial cells of the bile ducts generally expressed moderate levels of IFNAR2 (Figure 3B, bottom). Accordingly, the latter level of staining was used as an inner control within the sample, which was designated arbitrarily as intensity level 1. IFN-*α*/type 2 interferon receptor expression was noted in 69.2% (nine out of 13) of cases. The median progression-free survival rate was 12.6 months for IFNAR2-positive cases, 1.7 months for IFNAR2-negative cases. The time-to-progression survival rates at 1, 2, and 3 years of IFNAR2-positive patients (*n*=9) were significantly higher than the respective rates of IFNAR2-negative patients (*n*=4) (*P*=0.0002, Figure 4A). The median overall survival rate was 24.4 months for IFNAR2-positive cases, 3.4 months for IFNAR2-negative cases. The overall survival rates at 1, 2, and 3 years of IFNAR2-positive patients (*n*=9) (88.9, 55.6, and 22.2%, respectively) were significantly higher than the respective rates (0, 0, and 0%) of IFNAR2-negative patients (*n*=4) (*P*<0.0001 each, Figure 4B).

### Clinical and pathological correlations

**Figure 5:**
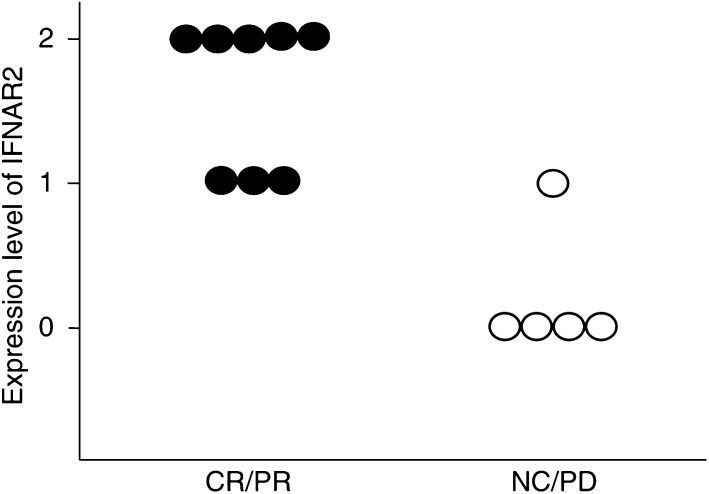
Correlation between IFNAR2 expression and efficiency of IFN-*α*/5-FU combination therapy. In eight objective responders, five cases showed intensity 2, three cases intensity 1, and none showed intensity 0. In five nonresponders, one case showed intensity 1, four cases intensity 0, and none showed intensity 2. There was a significant correlation between IFNAR2 expression and response to therapy.

Finally, we compared the responders (CR/PR) (*n*=8) with the nonresponders (NC/PD) (*n*=5) in terms of serum AFP (within normal range; <5), serum PIVKA-II (normal range; <45), Child–Pugh score, OKUDA score, CLIP score, and IFNAR2 expression by univariate analysis (Table 3). Serum AFP, PIVKA-II, Child–Pugh score, OKUDA score, and CLIP score did not correlate with the response to combination therapy. On the other hand, IFNAR2 expression correlated significantly with the response to IFN-*α*/5FU combination therapy (*P*=0.0070). Thus, the expression level of IFNAR2 was the sole factor that influenced the response to the combination therapy. The correlation between IFNAR2 expression and response to therapy is shown in Figure 5.

## DISCUSSION

In this study, we showed the beneficial effects of IFN-*α*/5-FU combination therapy in patients with multiple lesions and tumour thrombi in the major branches of the portal vein (Vp3 or 4), as our second report on this treatment. The efficacy of such treatment was 43.6% in our patients with highly advanced HCC, which was almost similar to the previous report of patients with the same stage HCC (Urabe *et al*, 1998). The prognosis of such patients is extremely poor and survival is generally limited to a few months after diagnosis, despite multimodal therapies even in cases suitable for surgical resection (Poon *et al*, 2003). The combination treatment of IFN-*α* and 5-FU markedly decreased tumour size and levels of tumour markers with an encouraging response rate and prolonged survival time in the responders. Furthermore, the clinical response completely reflected the survival benefits, as shown in Figures 1 and 2. On the other hand, almost all nonresponders died within 6 months. No response to the combination therapy was seen in 56.4% (31 out of 55) of our patients in this study. To advance the effect of IFN-*α*/5-FU combination therapy and to increase the response rate, it is necessary to investigate the mechanism of IFN-*α*/5-FU combination therapy.

Several mechanisms for the anticancer effects of IFN-*α*, with or without 5-FU, have been proposed. In general, both agents have antitumour properties, even when used alone. These effects can be direct and/or indirect (immunological) antitumour effects. The direct antitumour effects include cell damage (Grander *et al*, 1993), upregulation of cancer antigen (Guadagni *et al*, 1989), and delayed action on the cell cycle (Kimchi, 1992). On the other hand, the indirect antitumour actions include activation of natural killer cells (Ortaldo *et al*, 1983), T cell system (Brinkmann *et al*, 1993), and macrophages (Uno *et al*, 1985). It is also possible that both IFN-*α* and 5-FU reinforce the antitumour action of each other or have additive effects. *In vitro* experiments showed that IFN-*α* induces cyclin-dependent kinase inhibitors involved in G1/G0 arrest (Sangfelt *et al*, 1999). Interferon-*α* may also exert its antitumour effect indirectly via the immune system since IFN-*α* is known to augment T-cell cytotoxicity (Lindahl *et al*, 1972; Trinchieri *et al*, 1978). Recently, we reported that the modulation of tumour necrosis factor-related apoptosis-inducing ligand (TRAIL) receptor-mediated cytotoxic pathway could partially contribute to the anti-HCC effect of IFN-*α*/5-FU combination therapy (Yamamoto *et al*, 2004). In addition, several experimental studies demonstrated that IFN-*α* enhanced the cytotoxic effect of 5-FU in various cultured malignant cells (Wadler and Schwartz, 1990; Schwartz *et al*, 1992). The upregulation of 5-FU activities combined with IFN-*α* was also shown by our laboratories (Damdinsuren *et al*, 2003).

Moreover, IFN-*α* suppressed the proliferation of all IFNAR2-positive HCC cell lines *in vitro* through mechanisms related to apoptosis or inhibition of cell cycle (Yano *et al*, 1999). The importance of IFNAR2 expression for the anticancer effect of IFN-*α*/5-FU was shown in such situation in our previous report (Eguchi *et al*, 2000). These findings suggest that the antineoplastic effects of IFN-*α* are likely to be mediated through its high-affinity membrane type I receptor, IFNAR2 (Darnell *et al*, 1994). In this regard, IFNAR2 expression in HCC tissues may be a useful predictor of response to such therapy and thus distinguish between responders and nonresponders to IFN-*α*/5-FU combination therapy.

We previously validated the suitability of IFNAR2 antibody (OCT4813) used in the present study, by the agreement between the results of immunohistochemistry and Western blot analysis; when expression of IFNAR2 protein was examined after cell fractionation in a patient positive for IFNAR2, the long and short forms of IFNAR2 were predominant in the cell membrane fraction while the soluble form was observed only in the cytosol fraction (Kondo *et al*, 2000). This result demonstrated that the immunohistochemically evident expression of IFNAR2 correlated with the expression of the active form of IFNAR2 (Yatsuhashi *et al*, 1999). The reliability of this antibody was also established in two other reports (Takayama *et al*, 2000; Fujiwara *et al*, 2003). Based on these results, we investigated the relationship between IFNAR2 expression and the effect of IFN-*α*/5-FU combination therapy using immunohistochemical analysis. Our results showed good correlation between the effect of such therapy and expression level of IFNAR2.

Several markers for prediction of tumour recurrence and prognosis have been identified. Levy and Sherman (2002) reported that the CLIP classification for HCC is easier to implement and more accurate than the OKUDA classification. In addition, Koike *et al* (2001) suggested that serum PIVKA-II level is the most useful predisposing clinical parameter for the development of portal vein invasion. To investigate the role of these clinical parameters, AFP, PIVKA-II, OKUDA score, and CLIP score were used in the present study to assess the clinical effects of IFN-*α*/5-FU combination therapy. The results showed that the expression of IFNAR2 was the only significant predictor of clinical outcome of IFN-*α*/5-FU combination therapy; no significant correlation was found with other factors, including serum AFP, serum PIVKA-II, Child–Pugh score, Okuda Score, and CLIP score. Moreover, survival analysis showed the significant role of IFNAR2 expression on prognosis; IFNAR2-positive cases had better prognosis than negative cases. These results suggest that the expression of IFNAR2 could be a potentially useful predictor of the response to IFN-*α*/5-FU combination therapy. In our recent report using microarray analysis, several genes concerned with IFN signalling transduction were found useful for molecular prediction of response to IFN-*α*/5-FU combination therapy in advanced HCC (Kurokawa *et al*, 2004).

Two issues should be discussed in relation to our results. First, IFN-*α*/5-FU combination therapy had no effect in IFNAR2-negative cases. Upregulation of IFNAR2 may be considered in order to induce a better response to the therapy in such cases. This proposition is based on the results of early studies, in addition to those of the present study. For example, Wagner *et al* (2004) showed that LOX, MDA231, MT1, and HT1080 cell lines transfected with IFNAR2c demonstrated a marked increase in their IFN-dependent antiproliferative response. Applying this method to HCC cells, IFNAR1/2 transfection might lead to overexpression of IFNAR2 and IFN-*α*-induced cell apoptosis. One recent study involving *in vitro* experiments showed that IFNAR2 gene transfer is effective in augmenting the biological activity of IFN-*α*/5-FU combination therapy in human HCC (Kondo *et al*, 2005). Thus, IFNAR2 gene transfection might enhance the response to IFN-*α*/5-FU combination therapy in IFNAR2-negative patients.

Second, not all IFNAR2-positive cases benefited from IFN-*α*/5-FU combination therapy. In such patients, increasing the doses or modifying the combination therapy (e.g. addition of other antitumour agents) might increase the response rate. Other parameters, apart from the expression of IFNAR2, might be important and necessary for the response to IFN-*α*/5-FU combination therapy. A recent report showed that transcription of the p53 gene is induced by IFN-*α*, accompanied by an increase in p53 protein level (Takaoka *et al*, 2003). The study suggested that p53 gene might be an important predictor of IFN-*α* therapy for HCV liver cirrhosis and HCC. In addition to IFNAR2 immunohistochemistry, p53 sequence analysis may identify other factors that could predict the response to IFN-*α*/5-FU combination therapy. In this regard, Yano *et al* (1999) used HCC cell lines and showed that normal p53 gene expression is not necessary for IFN-*α*-induced apoptosis. Thus, further studies are required to determine the importance of p53 mutation on the response to IFN-*α*/5-FU combination therapy.

In conclusion, we demonstrated the efficacy of IFN-*α*/5-FU combination therapy for patients of advanced HCC with tumour thrombi in major branches of the portal vein. We also showed that the clinical response to such therapy correlated significantly with the expression of IFNAR2 in HCC.

## Figures and Tables

**Table 1:** Patients characteristics

	**(*n*=55)**
*Age (years)*	
Mean±s.d.	58.2±9.4

*Gender*	
Male/female	49/6

*Hepatitis virus*	
HBsAg+, HCVAb−	19
HBsAg−, HCVAb+	24
HBsAg+, HCVAb+	5
HBsAg−, HCVAb−	5
Unknown	2

*Granulocytes (μl* ^−1^ *)*	
Mean±s.d.	5310±1958

*Platelets (* × *10^4^ μl*^−1^*)*	
Mean±s.d.	15.3±6.7

*Serum albumin (g dl* ^−1^ *)*	
Mean±s.d.	3.22±0.44

*Serum bilirubin (mg dl* ^−1^ *)*	
Mean±s.d.	0.94±0.36

*Prothrombin time (s)*	
Mean±s.d.	17.9±2.0

*Child–Pugh classification*	
A	19
B	31
C	4
Unknown	1

*AFP (ng ml* ^−1^ *)*	
<5	2
⩾5	52
Unknown	1

*PIVKA-II (mAU ml* ^−1^ *)*	
<40	2
⩾40	52
Unknown	1

*Tumour size (cm)*	
Mean±s.d.	6.4±3.2

HbsAg=hepatitis B serum antigen; HCVAb=hepatitis C viral antibody; AFP=alpha-fetoprotein; PIVKA-II= protein induced by vitamin K absence or antagonist-II.

**Table 2:** Adverse effects

	**Patients (*n*=55)**			
	**Grade 1**	**Grade 2**	**Grade 3**	**Grade 4**
*Haematological*				
Leukopenia	7	11	3	0
Anemia	0	0	1	0
Thrombocytopenia	7	10	5	0

*Nonhaematological*				
Fever	52	3	0	0
Chilling sense	52	0	0	0
Nausea	3	0	0	0
Diarrhoea	2	0	0	0
Gastric ulcer	0	1	0	0
Flu-like syndrome	55	0	0	0
Skin reaction	3	0	0	0
General fatigue	14	0	0	0
Depression	2	0	0	0

**Table 3:** Univariate analysis for efficiency of IFN-*α*/5-FU combination therapy based on serum AFP, serum PIVKA-II, Child–Pugh score, CLIP score, and IFNAR2 expression

	**CR/PR (*n*=24)**	**NC/PD (*n*=31)**	***P*-value**
*Age*			
<60	12	15	>0.9999
⩾60	12	16	

*Gender*			
Male	21	28	>0.9999
Female	3	3	

*Child–Pugh*			
A	8	11	>0.9999
B,C	15	20	

*AFP*			
<400	14	15	0.5919
⩾400	10	15	

*PIVKA-II*			
<65	3	1	0.3123
⩾65	21	29	

*Okuda*			
1	19	23	0.3270
2,3	3	8	

*CLIP*			
0,1,2,3	12	13	0.7843
4,5,6	12	17	

*IFNAR*			
Negative	0	4	0.0070
Positive	8	1	

CR=complete response; PR=partial response; NC=no change; PD=progressive disease; AFP=alpha-fetoprotein; PIVKA-II=protein induced by vitamin K antagonists or absence.
